# U-shaped relationship between the triglyceride glucose index and the risk of incident diabetes among MASLD adults: a retrospective cohort study

**DOI:** 10.3389/fendo.2025.1516187

**Published:** 2025-08-22

**Authors:** Changchun Cao, Xiaohua Zhang, Yong Han, Haofei Hu, Yulong Wang

**Affiliations:** ^1^ Department of Rehabilitation, Shenzhen Second People’s Hospital, Shenzhen Second People’s Hospital Dapeng Hospital, Shenzhen, Guangdong, China; ^2^ Department of Emergency, Shenzhen Second People’s Hospital, The First Affiliated Hospital of Shenzhen University, Shenzhen, Guangdong, China; ^3^ Department of Nephrology, Shenzhen Second People’s Hospital, The First Affiliated Hospital of Shenzhen University, Shenzhen, Guangdong, China; ^4^ Department of Rehabilitation, Shenzhen Second People’s Hospital, The First Affiliated Hospital of Shenzhen University, Shenzhen, Guangdong, China

**Keywords:** metabolic dysfunction-associated steatotic liver disease, type 2 diabetes, triglyceride, triglyceride glucose index, insulin resistance

## Abstract

**Background:**

Previous research has indicated that the triglyceride glucose index (TyG-i) may serve as a potential risk factor for type 2 diabetes (T2D). However, there is a paucity of studies addressing the relationship between TyG-i and T2D, specifically in patients with metabolic dysfunction-associated steatotic liver disease (MASLD). Consequently, this longitudinal study aims to investigate the association between TyG-i and the onset of T2D in a cohort of Japanese adults with MASLD.

**Methods:**

This retrospective cohort study included a total of 2,507 subjects diagnosed with MASLD. To evaluate the association between the TyG-i and the risk of developing T2D, Cox proportional hazards regression models were employed to estimate hazard ratios (HR) along with 95% confidence intervals (CI). Additionally, nonlinear associations between them were investigated utilizing restricted cubic spline models.

**Results:**

During a mean follow-up period of 6.00 years, a total of 204 adults with MASLD developed T2D. After adjusting for potential confounding factors, elevated TyG-i was found to be independently associated with an increased risk of developing T2D (HR: 1.48, 95% CI: 1.05-2.09, P = 0.0256). Additionally, a U-shaped relationship between the TyG-i and the incidence of T2D was identified. A significant negative association was observed between TyG-i and T2D risk when TyG-i levels were below 7.94 (HR: 0.21, 95%CI: 0.07-0.66, P = 0.0072). Conversely, TyG-i values exceeding the threshold were positively correlated with T2D risk (HR: 1.76, 95% CI: 1.23-2.52, P = 0.0020).

**Conclusion:**

A U-shaped association was identified between baseline TyG-i and the incidence of T2D in a Japanese population with MASLD. This inflection point in TyG-i serves as a valuable clinical indicator to differentiate individuals at lower versus higher risk of developing T2D. These findings indicate that maintaining TyG-i near the inflection point may be beneficial in reducing the risk of developing diabetes in patients with MASLD.

## Introduction

Metabolic dysfunction-associated steatotic liver disease (MASLD) represents the most prevalent chronic liver disorder globally (1–3), impacting approximately 32% of the world’s population (4). This condition is marked by excessive lipid deposits in the liver, which can progress to inflammation and liver injury. Without intervention, these changes can advance to liver cirrhosis and potentially hepatocellular carcinoma (3, 5).

MASLD is linked not only to elevated liver-related health issues and mortality rates but also to an increased likelihood of developing cardiovascular diseases, type 2 diabetes (T2D), and overall mortality (1, 6–8). Research indicates that MASLD may act as a precursor to or exacerbate the onset of T2D (1, 9). Recent epidemiological investigations reveal that individuals diagnosed with MASLD face a two-fold greater risk of developing diabetes compared to those without the disease (10). Consequently, it is crucial to comprehend the fundamental risk factors that lead to glucose dysregulation in patients with MASLD, as this knowledge could guide the formulation of effective preventive measures against the onset of diabetes.

The triglyceride glucose index (TyG-i) has emerged as a significant biomarker for evaluating insulin resistance and predicting diabetes risk (11, 12). This index is derived from fasting triglyceride and glucose levels, offering a straightforward yet effective measure of metabolic health. Numerous studies have established substantial correlations between TyG-i and various health outcomes. Recent research has identified associations between TyG-i and conditions such as MASLD, cardiovascular disease, gestational diabetes, prediabetes, T2D, and all-cause mortality (13–16). Despite the increasing evidence linking TyG-i to T2D risk within general populations, its specific relationship with T2D among individuals with MASLD remains inadequately explored. Given the shared pathophysiological mechanisms of insulin resistance and dyslipidemia that characterize both MASLD and T2D, investigating TyG-i within the context of MASLD presents a unique opportunity to clarify its role as an early predictor of diabetes onset. Consequently, this retrospective study aims to examine the longitudinal association between TyG-i and the development of T2D among individuals with MASLD.

## Methods

### Data source and study participants

The data utilized in our research were obtained from the NAGALA database (17), which is hosted on the Dryad Data Platform. According to the service terms of the Dryad database, this dataset is available for analysis to support the exploration of new research hypotheses. The NAGALA database is a population-based longitudinal cohort study conducted at Murakami Memorial Hospital in Gifu Prefecture, Japan, spanning from 1994 to 2016 (17).

**Figure 1:**
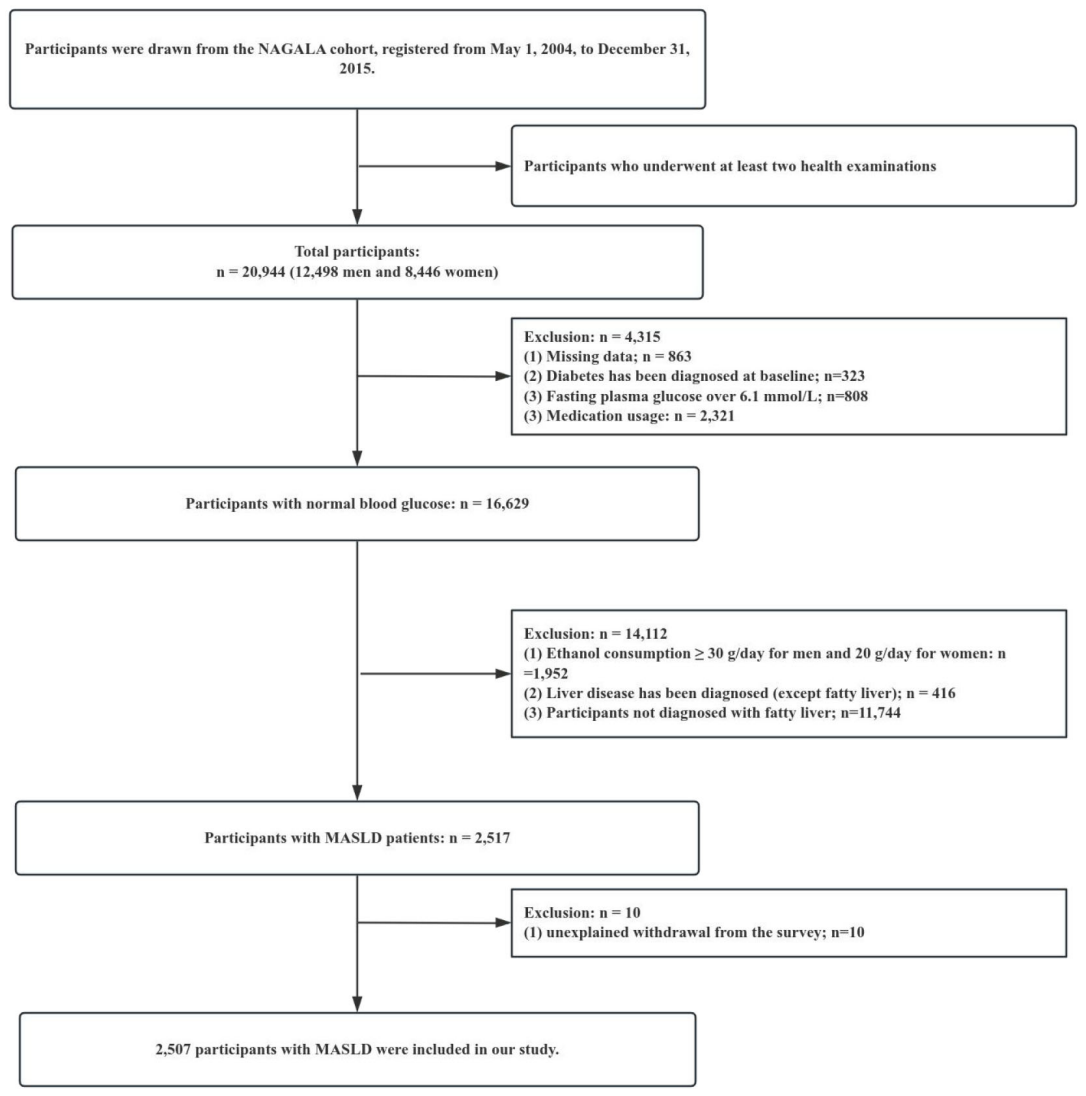
Study population.

Participants in this study underwent a minimum of two physical examinations. In the initial study conducted by Okamura T et al. (17), medical data were extracted from a total of 20,944 participants. The exclusion criteria were as follows: (1) excessive alcohol consumption at baseline, defined as ≥30 g/day for females and ≥20 g/day for males (n = 1,952); (2) pre-existing liver disease (n = 416); (3) use of medications (n = 2,321); (4) missing data (n = 863); (5) a diagnosis of diabetes at baseline or fasting plasma glucose (FPG) levels exceeding 6.1 mmol/L (n = 1,131); and (6) participants not diagnosed with fatty liver disease (n = 11,744). Ultimately, our study included 2,507 participants with MASLD. The selection process for all participants is illustrated in **Figure 1**. Ethical approval for this research was obtained from the Clinical Research Ethics Committee of Shenzhen Second People’s Hospital Dapeng New District Nan’ao Hospital. Additionally, the study was conducted in accordance with the principles set forth in the Declaration of Helsinki, ensuring adherence to all pertinent guidelines and regulatory requirements. To ensure data confidentiality, all personal identifiers were removed and the datasets were anonymized before analysis. Data were stored in secure servers with access restricted to authorized study personnel only. Throughout the study, data handling adhered to applicable data protection laws and institutional policies, thereby safeguarding participant privacy and confidentiality.

### Covariates

We choose covariates using clinical expertise and previous research results (14, 18–24). The covariates included (1) continuous variables: age, systolic blood pressure (SBP), diastolic blood pressure (DBP), body mass index (BMI), alcoholic intake, high-density lipoprotein cholesterol (HDL-C), total cholesterol (TC), alanine aminotransferase (ALT), gamma-glutamyl transferase (GGT), aspartate aminotransferase (AST), glycosylated hemoglobin (HbA1c), and FPG; (2) categorical variables: sex, smoking status, and exercise habits. The initial investigation employed a standardized self-administered questionnaire to collect comprehensive information on participants’ medical backgrounds and lifestyle habits. Past-smoker is defined as individuals who have a history of smoking but has not engaged in smoking behavior within the 12 months preceding their enrollment in the study. Trained professionals conducted precise anthropometric measurements, including body mass and stature. The original study team obtained Laboratory test results using consistent procedures under controlled conditions.

### TyG-i

The TyG-i was determined by applying the formula: Ln[FPG (mg/dL))×(TG (mg/dL)/2) (14).

### Diagnosis of incident T2D

T2D was defined as having a self-reported history, HbA1c ≥ 6.5%, or FPG≥7.0 mmol/L (25).

### Statistical analysis

Statistical analyses were conducted utilizing Empower-Stats. Participant baseline characteristics were assessed across quartiles of the TyG-i. Data with normal distribution are expressed as means with standard deviations, whereas non-normally distributed data are reported as medians accompanied by interquartile ranges. Categorical variables underwent analysis via the chi-square test, while continuous variables were evaluated using Student’s t-test for normally distributed data and the Mann-Whitney U test for data not following a normal distribution.

The association between the TyG-i and T2D risk was evaluated through three Cox regression models. DBP was omitted from the final multivariate Cox proportional hazards regression model following the collinearity assessment (**Supplementary Table S1**). Model 1 represents the unadjusted analysis. Model 2 incorporates adjustments for demographic and lifestyle variables, including sex, age, exercise habits, smoking status, alcoholic intake, and SBP. Model 3 further extends the adjustments to include biochemical parameters: ALT, GGT, AST, TC, HDL-C, and HbA1c. Throughout the study, we documented hazard ratios (HR) and 95% confidence intervals (CI). To explore the nonlinear association between the TyG-i and T2D risk, restricted cubic spline curves were generated based on Model 3 in the Cox proportional hazard analysis. This approach allows flexible modeling of the dose-response relationship without assuming linearity. When nonlinearity was detected, the inflection point was identified using a recursive algorithm designed to find the value of TyG-i at which the risk pattern changes. Subsequently, a two-piecewise Cox proportional hazards regression model was constructed on either side of the inflection point, enabling estimation of separate hazard ratios for TyG-i below and above this threshold to better characterize the relationship.

Hypertension and advanced age are well-documented risk factors for diabetes, as established by numerous scholarly studies. To assess the robustness of the relationship between TyG-i and T2D risk, sensitivity analyses were performed, excluding subjects with hypertension (SBP≥140 mmHg or DBP≥ 90 mmHg) or elderly (age≥60 years). In addition, to address potential residual confounding inherent in observational studies, the E-value was calculated as a sensitivity analysis metric. The E-value quantifies the minimum strength of association that any unmeasured confounder would need to possess with both the TyG-i and the incidence of diabetes, beyond the measured covariates, in order to completely explain away the observed association. This provides a quantitative measure of the robustness of our findings against unmeasured confounding.

A stratified analysis including age (≤60 years old or >60 years), gender, hypertension (DBP ≥90 mmHg or SBP ≥140 mmHg), BMI (<25, ≥25 kg/m^2^), alcoholic intake (0, >0 g/wk), smoking status, and exercise habits was conducted to evaluate the potential effects of covariates. Statistical significance was defined as a two-tailed P value of < 0.05.

## Results

### Characteristics of the study population

The present study encompassed 2,507 participants diagnosed with MASLD, with an average age of 44.78 ± 8.33 years, of which 80.93% were male. Over an average follow-up duration of 6.00 years, 204 participants (8.14%) developed T2D. Participants were categorized into quartiles based on their TyG-i values: Q1 (TyG-i ≤ 8.21), Q2 (8.21 < TyG-i ≤ 8.58), Q3 (8.58 < TyG-i ≤ 8.94), and Q4 (TyG-i > 8.94) (**Table 1**). Individuals in the highest TyG-i quartile demonstrated higher levels of SBP, DBP, BMI, GGT, AST, ALT, TG, TC, age, alcoholic intake, HbA1c, and FPG, as well as a greater proportion of male participants and smokers. Additionally, these individuals exhibited lower levels of HDL-C.

**Table 1:** The characteristics of participants and incidence rate of diabetes.

TyG-i	Q1 (≤8.21)	Q2 (8.21 to ≤8.58)	Q3 (8.58 to ≤8.94)	Q4 (>8.94)	P-value
Participants	627	625	628	627	
Sex					<0.001
Female	188 (29.98%)	143 (22.88%)	88 (14.01%)	59 (9.41%)	
Male	439 (70.02%)	482 (77.12%)	540 (85.99%)	568 (90.59%)	
Age(years)	44.74 ± 8.62	44.91 ± 8.45	45.03 ± 8.18	44.45 ± 8.07	0.641
Alcoholic intake (g/wk)	1 (0-18)	1 (0-36)	1 (0-44)	4.2 (1-60)	<0.001
Smoking status					<0.001
Never-smoker	350 (55.82%)	316 (50.56%)	269 (42.83%)	250 (39.87%)	
Past-smoker	152 (24.24%)	169 (27.04%)	161 (25.64%)	157 (25.04%)	
Current-smoker	125 (19.94%)	140 (22.40%)	198 (31.53%)	220 (35.09%)	
Exercise habits					0.469
No	528 (84.21%)	528 (84.48%)	529 (84.24%)	545 (86.92%)	
Yes	99 (15.79%)	97 (15.52%)	99 (15.76%)	82 (13.08%)	
SBP (mmHg)	120.61 ± 14.05	122.92 ± 15.19	123.66 ± 14.36	126.43 ± 15.14	<0.001
DBP (mmHg)	75.57 ± 9.87	77.39 ± 10.36	78.17 ± 9.60	80.11 ± 10.41	<0.001
BMI (kg/m^2^)	24.81 ± 2.98	25.37 ± 3.44	25.80 ± 3.13	26.00 ± 2.81	<0.001
ALT (IU/L)	24 (18-32.50)	25 (19-35)	28 (21-40)	31 (23-45)	<0.001
AST (IU/L)	19 (16-24)	20 (16-25)	21 (17-26)	22 (18-28)	<0.001
GGT (IU/L)	18 (14-25)	22 (16-30)	24 (17-35)	29 (21-41)	<0.001
HDL-C (mg/dL)	52.64 ± 12.08	47.28 ± 10.28	43.77 ± 9.04	39.69 ± 8.18	<0.001
TG (mg/dL)	60 (49-69)	93 (84-101)	131 (120-142)	203 (176-252)	<0.001
TC (mg/dL)	196.03 ± 32.42	205.70 ± 29.39	215.83 ± 32.94	224.13 ± 32.64	<0.001
HbA1c (%)	5.28 ± 0.32	5.29 ± 0.34	5.30 ± 0.34	5.33 ± 0.33	0.033
FPG (mg/dL)	95.15 ± 6.75	96.97 ± 6.46	97.40 ± 6.32	99.17 ± 6.06	<0.001

Values are presented as n (%) or mean ± SD or median (quartile).

TyG-i, triglyceride glucose index; BMI, body mass index; SBP, systolic blood pressure; DBP, diastolic blood pressure; ALT, alanine aminotransferase; AST, aspartate aminotransferase; GGT, gamma-glutamyl transferase; HDL-C, high-density lipoprotein cholesterol; TC, total cholesterol; TG, triglycerides; HbA1c, hemoglobin A1c; FPG, fasting plasma glucose.

### The incidence rate of T2D

**Figure 2:**
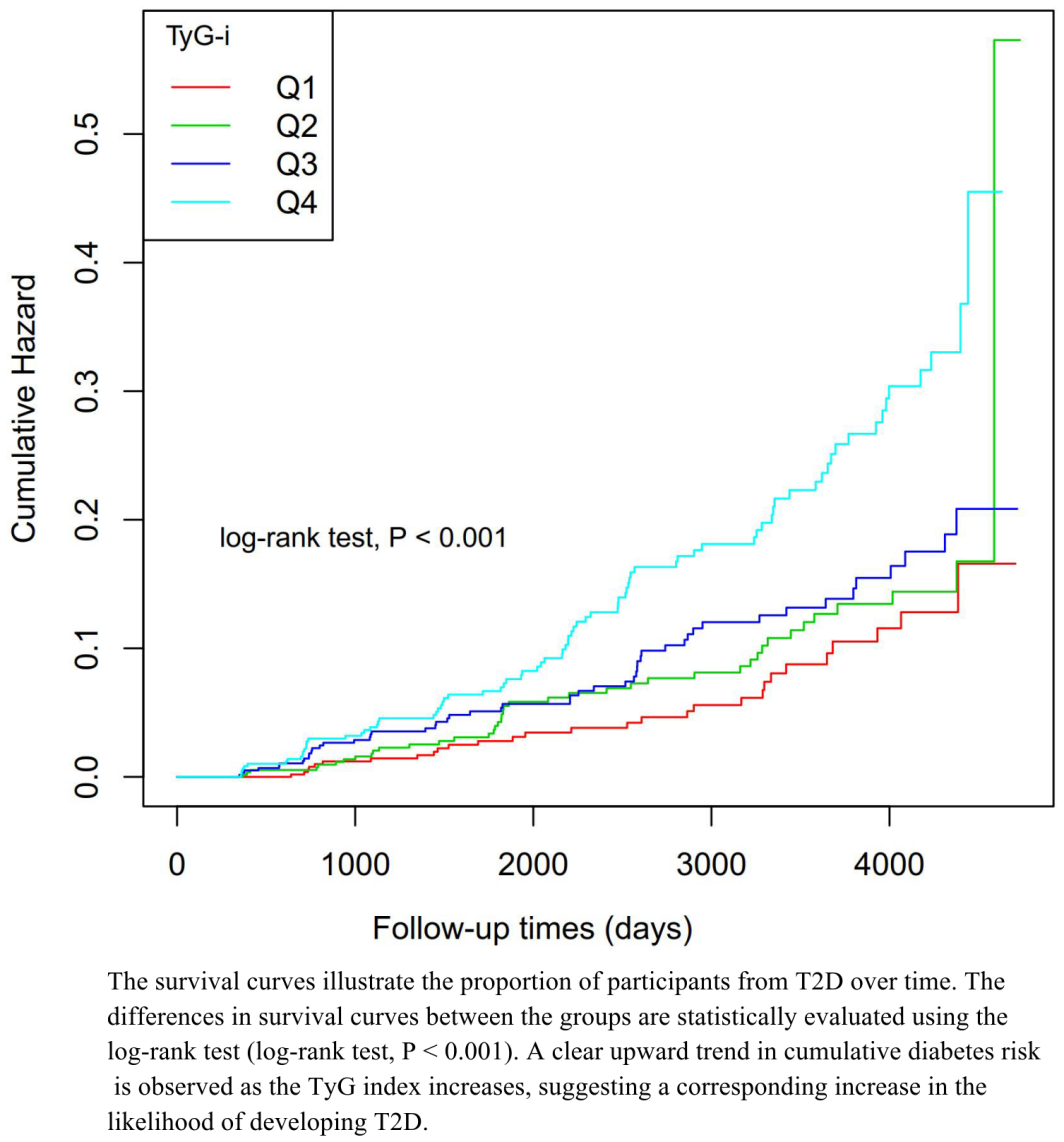
Kaplan–Meier event-free survival curve in females. Kaplan–Meier analysis of incident diabetes based on TyG-i quartiles (log-rank, P < 0.0001).

**Table 2** further illustrates that during the follow-up period, 373 individuals developed T2D, corresponding to overall incidence rates of 4.63% (95%CI: 2.98%-6.27%), 6.56% (95%CI: 4.61%-8.51%), 8.12% (95%CI: 5.98%-10.26%), and 13.24% (95%CI: 10.58%-15.90%) across the first, second, third, and fourth TyG-i groups, respectively. The cumulative incidence rates per 100,000 person-years were 1,356.01 for the total study population and 792.88, 1,082.48, 1,326.71, and 2,210.45 for the first, second, third, and fourth TyG-i groups, respectively. The data indicate that higher TyG-i levels are associated with increased incidence and cumulative prevalence of T2D. Participants positioned within the higher TyG-i quartiles exhibited significantly elevated incidence rates of T2D. These findings are corroborated by the Kaplan-Meier curve illustrating cumulative hazard, as presented in **Figure 2**.

**Table 2:** Incidence rate of incident diabetes.

TyG-i	Participants (n)	Diabetes events (n)	Cumulative incidence (95% CI) (%)	Per 100,000 person-year
Total	2507	204	8.14 (7.07-9.21)	1,356.01
Q1	627	29	4.63 (2.98-6.27)	792.88
Q2	625	41	6.56 (4.61-8.51)	1,082.48
Q3	628	51	8.12 (5.98-10.26)	1,326.71
Q4	627	83	13.24 (10.58-15.90)	2,210.45
P for trend			<0.001	<0.001

TyG-i, triglyceride glucose index; CI, confidence interval; T2D, type 2 diabetes.

### The results of the association between TyG-i and T2D risk

Since the TyG-i satisfied the proportional hazards assumption, the relationship between TyG-i and the risk of T2D was assessed using the Cox proportional hazards regression model. The outcomes from the adjusted multivariable Cox proportional hazards regression models are detailed in **Table 3**. An elevated TyG-i value was linked with the occurrence of T2D. In Models 1, 2, and 3, employing continuous TyG-i, significant associations between TyG-i and T2D risk were observed (Model 1: HR: 2.03, 95%CI: 1.57-2.63, P<0.0001; Model 2: HR: 2.13, 95%CI: 1.62-2.79, P<0.0001; Model 3: HR: 1.48, 95%CI: 1.05-2.09, P=0.0256). Furthermore, in Model 3, the highest quartile of TyG-i exhibited a 56% increased risk of T2D (HR: 1.56, 95%CI: 0.92-2.64) compared to the lowest quartile.

**Table 3:** Relationship between TyG-i and incident diabetes in different models.

Variable	Model 1 (HR, 95%CI, P)	Model 2 (HR, 95%CI, P)	Model 3 (HR, 95%CI, P)
TyG-i	2.03 (1.57, 2.63) <0.0001	2.13 (1.62, 2.79) <0.0001	1.48 (1.05, 2.09) 0.0256
TyG-i (quartile)			
Q1	Ref	Ref	Ref
Q2	1.33 (0.83, 2.15) 0.2349	1.33 (0.82, 2.14) 0.2455	1.01 (0.62, 1.66) 0.9545
Q3	1.63 (1.03, 2.57) 0.0361	1.63 (1.03, 2.60) 0.0386	1.28 (0.77, 2.12) 0.3375
Q4	2.76 (1.81, 4.21) <0.0001	2.82 (1.82, 4.37) <0.0001	1.56 (0.92, 2.64) 0.0967
P for trend	<0.0001	<0.0001	0.0403

Model 1: we did not adjust for any covariants.

Model 2: we adjusted for sex, age, alcoholic intake, smoking status, exercise habits, and SBP.

Model 3: we adjusted for sex, age, alcoholic intake, smoking status, exercise habits, SBP, ALT, AST, GGT, HDL-C, TC, and HbA1c.

HR, hazard ratio; CI, confidence interval; Ref, Reference; TyG-i, triglyceride glucose index.

### Sensitive analysis

To validate our results, we used extensive sensitivity analyses. Excluding participants with elevated blood pressure, we maintained a positive association between TyG-i and T2D (HR=1.45, 95% CI: 1.02-2.06, P=0.0380) (**Table 4**, Model 4). Similarly, excluding participants aged ≥60 years showed consistent results, with TyG-i remaining positively associated with T2D risk after adjusting for multiple covariates (HR=1.50, 95% CI: 1.03-2.17, P=0.0347) (**Table 4**, Model 5). Moreover, the calculated E-value of 2.32 surpasses the relative risk estimate of 1.78 attributed to both the TyG-i and plausible unmeasured confounding factors. This suggests that the impact of unidentified or unmeasured confounders on the detected association between TyG-i and T2D is probably limited.

**Table 4:** Relationship between TyG-i and incident T2D in different sensitivity analyses.

Exposure	Model 4 (HR, 95%CI, P)	Model 5 (HR, 95%CI, P)
TyG-i	1.45 (1.02, 2.06) 0.0380	1.50 (1.03, 2.17) 0.0347
TyG-i (quartile)		
Q1	Ref	Ref
Q2	1.04 (0.63, 1.72) 0.8763	1.21 (0.71, 2.06) 0.4947
Q3	1.25 (0.74, 2.09) 0.4024	1.36 (0.78, 2.39) 0.2760
Q4	1.54 (0.90, 2.63) 0.1135	1.76 (0.99, 3.15) 0.0548
P for trend	0.0568	0.0386

Model 4 was sensitivity analysis after excluding individuals with age≥60 years. We adjusted sex, age, alcoholic intake, smoking status, exercise habits, SBP, ALT, AST, GGT, HDL-C, TC, and HbA1c.

Model 5 was sensitivity analysis after excluding individuals with SBP≥140 mmHg or DBP≥ 90 mmHg. We adjusted sex, age, alcoholic intake, smoking status, exercise habits, SBP, ALT, AST, GGT, HDL-C, TC, and HbA1c.

HR, hazard ratios; CI, confidence; Ref, reference; TyG-i, triglyceride glucose index.

### The analyses of the non-linear association

**Table 5**, **Figure 3** demonstrate a U-shaped relationship between the TyG-i and T2D. The two-piecewise Cox regression model identified a turning point at a TyG-i value of 7.94 (P-value for the log-likelihood ratio test = 0.004). Below this turning point, TyG-i exhibited an inverse relationship with T2D risk (HR: 0.21, 95%CI: 0.07-0.66, P=0.0072). Conversely, when the TyG-i exceeded this turning point, a significant positive relationship with T2D risk was observed (HR: 1.76, 95% CI: 1.23-2.52, P=0.0020).

**Table 5:** The result of the two-piecewise Cox proportional hazards regression model.

Incident Diabetes	HR (95%CI)	P-value
Fitting model by standard linear regression	1.48 (1.05, 2.09)	0.0256
Fitting model by two-piecewise Cox proportional hazards regression		
The inflection point of TyG-i	7.94	
≤7.94	0.21 (0.07, 0.66)	0.0072
>7.94	1.76 (1.23, 2.52)	0.0020
P for the log-likelihood ratio test	0.004	

We adjusted sex, age, alcoholic intake, smoking status, exercise habits, SBP, ALT, AST, GGT, HDL-C, TC, and HbA1c.

HR, hazard ratios; CI, confidence; TyG-i, triglyceride glucose index.

**Figure 3:**
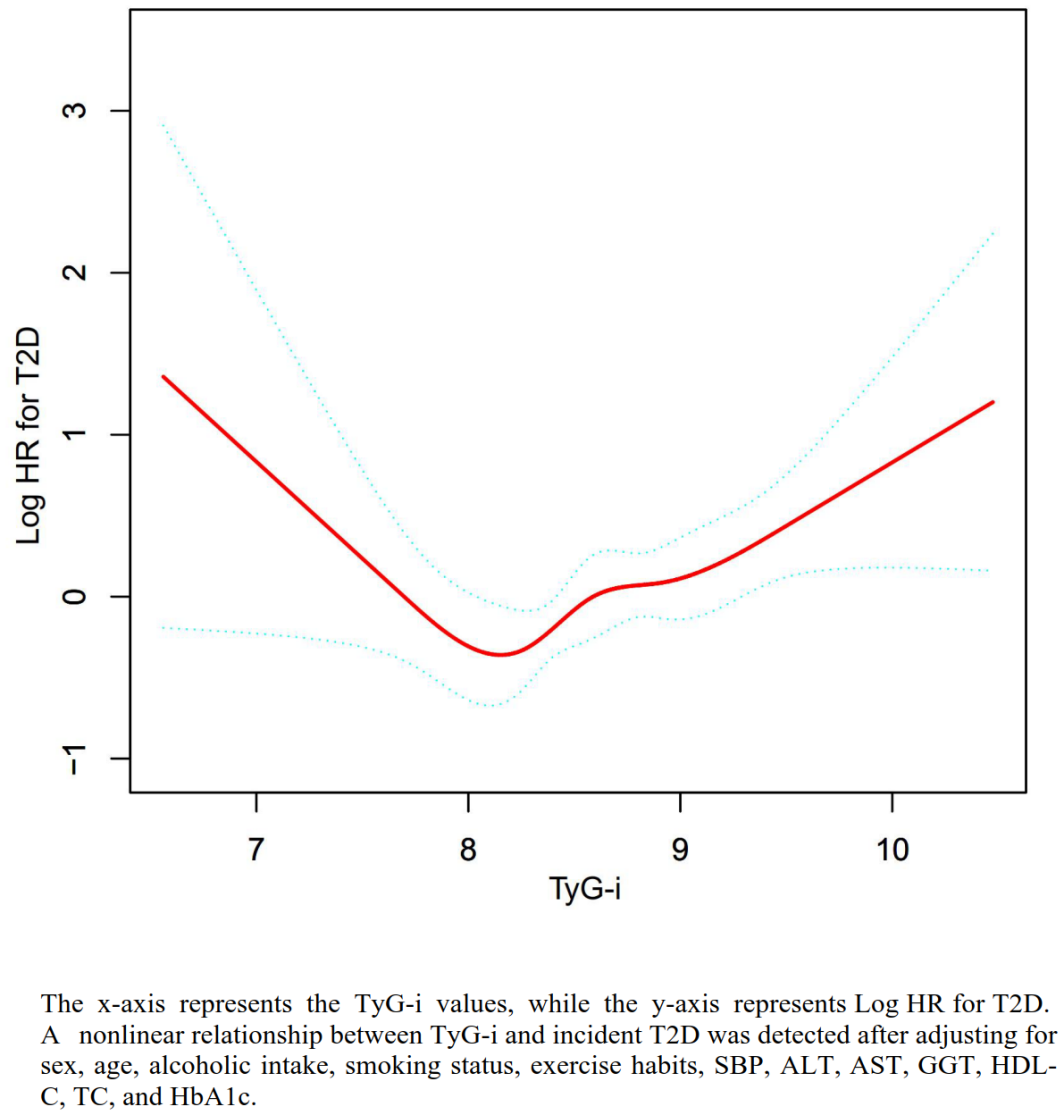
The nonlinear relationship between TyG-i and incident diabetes. The nonlinear relationship was detected after adjusting for sex, age, alcoholic intake, smoking status, exercise habits, SBP, ALT, AST, GGT, HDL-C, TC, and HbA1c.

### The results of the subgroup analysis

**Figure 4:**
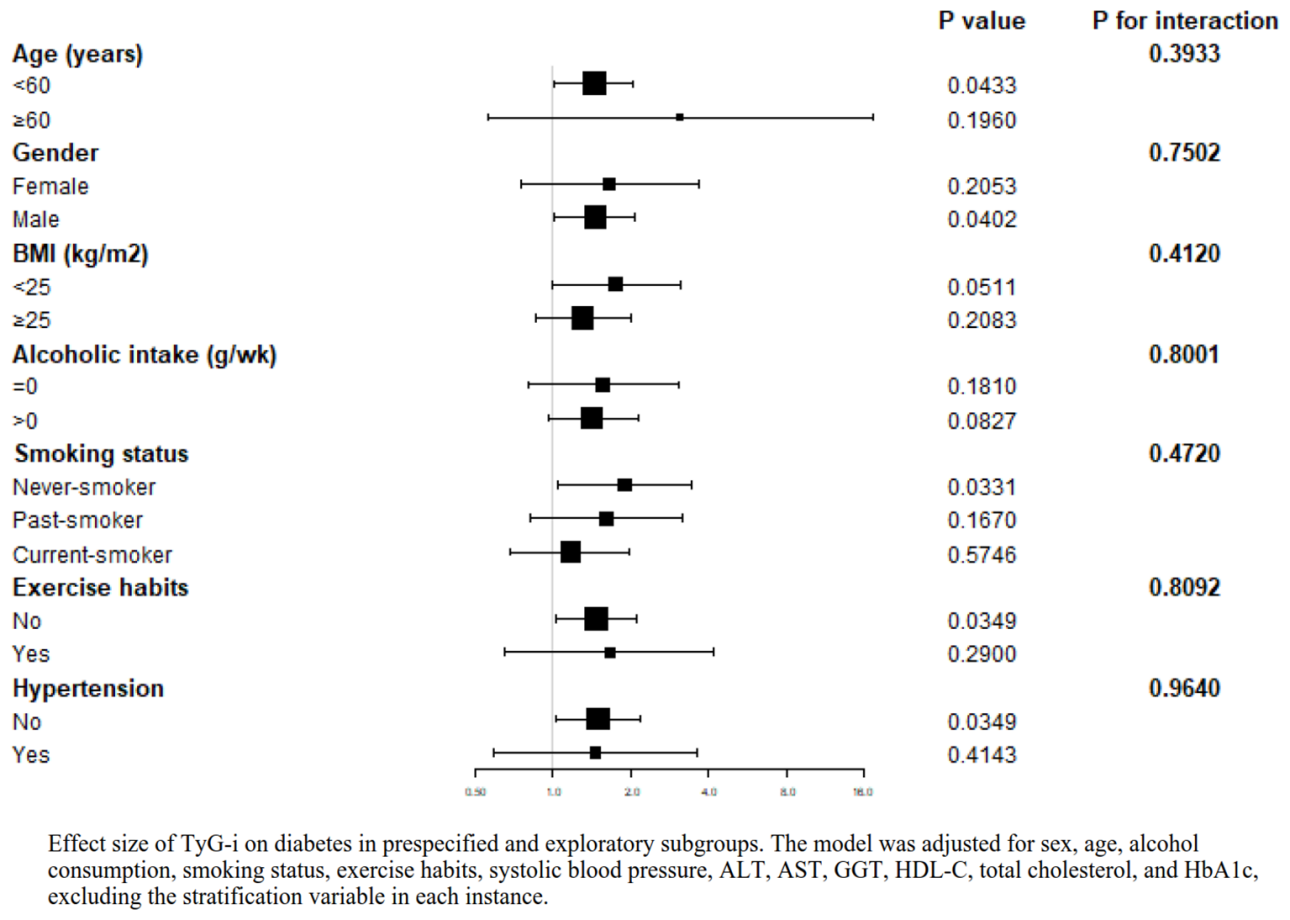
Effect size of TyG-i on diabetes in prespecified and exploratory subgroups. The model was adjusted for sex, age, alcohol consumption, smoking status, exercise habits, systolic blood pressure, ALT, AST, GGT, HDL-C, total cholesterol, and HbA1c, excluding the stratification variable in each instance.

**Figure 4** outlines the findings from subgroup analyses designed to identify potential modifiers in the association between the TyG-i and T2D. The analyses revealed no significant interactions with T2D across various subgroups, including age (P for interaction = 0.3933), smoking status (P for interaction = 0.4720), gender (P for interaction = 0.7502), exercise habits (P for interaction = 0.8092), BMI (P for interaction = 0.4120), hypertension (P for interaction = 0.9640), and alcohol intake (P for interaction = 0.8001).

## Discussion

In this retrospective cohort study involving 2,507 Japanese adults with MASLD, we identified a positive association between elevated TyG-i levels and the risk of T2D. Our findings further revealed a U-shaped relationship between TyG-i and an increased risk of T2D. Furthermore, sensitivity and subgroup analyses corroborated these results, reinforcing the robustness of our conclusions.

The TyG-i has been widely used as a surrogate for insulin resistance to predict the risk of metabolic diseases (26, 27). A meta-analysis that included 12 studies, including 105,365 participants, found that the TyG-i was positively associated with the risk of MASLD (OR: 2.84, 95%CI: 2.01-4.01) (28). In a longitudinal cohort study of 16,172 non-obese participants, individuals in the highest quartile of the baseline TyG-i had a 3.58-fold increased risk of developing MASLD relative to those in the lowest quartile (HR: 4.58, 95% CI: 3.48-6.02) (29). A meta-analysis encompassing 13 cohort studies with a total of 70,380 participants identified a significant and positive correlation between the TyG-i and T2D risk (HR: 2.44, 95% CI: 2.17-2.76) (30). In addition, a longitudinal cohort study that included 179541 Chinese adults found a positive nonlinear association between TyG-i and the risk of prediabetes and T2D after adjusting for confounders(HR: 1.67, 95%CI: 1.62-1.71, P< 0.001) (13). MASLD is a common chronic liver disease that is closely associated with metabolic syndrome (31). Past evidence has shown that the prevalence of diabetes is significantly increased in subjects with MASLD (8). However, there have been few studies discussing the relationship between TyG-i and T2D in the MASLD population. In our study, TyG-i was positively related to the risk of developing diabetes in people with MASLD when TyG-i > 7.94. Therefore, early intervention using the TyG-i may be effective in reducing the risk of diabetes in patients with MASLD.

Our research uncovered a U-shaped relationship between the TyG-i and T2D risk after controlling for confounders. Specifically, the analysis revealed that when TyG-i levels were below 7.94, there was a 79% decrease in the risk of T2D development for each one-unit increase in TyG-i. Conversely, a positive association was found between TyG-i and T2D risk when TyG-i levels exceeded 7.94. Understanding this U-shaped relationship is essential for identifying individuals exhibiting altered metabolic profiles across different TyG-i ranges. Those with values near the 7.94 inflection point may constitute a key population for targeted preventive interventions. Clinicians should closely monitor TyG-i as an early biomarker indicative of elevated T2D risk, particularly among patients with MASLD. Interventions aimed at sustaining TyG-i around the inflection point through lifestyle modifications—including dietary improvements, physical activity enhancement, and weight management—should be prioritized for individuals approaching this critical level. Such proactive measures could delay or prevent the progression from insulin resistance to overt T2D, thereby improving clinical outcomes. Additionally, the prospect of pharmacological strategies targeting the TyG-i warrants investigation. As the understanding of TyG-i’s metabolic implications advances, clinical trials designed to assess treatments that modulate TyG-i are necessary to expand therapeutic options for high-risk populations. From a public health perspective, our findings underscore the importance of recognizing TyG-i as a valuable marker in T2D risk stratification. Health professionals and policymakers should consider integrating TyG-i assessments into preventive care frameworks to optimize resource allocation and intervention efficacy. Furthermore, educational programs aimed at raising awareness of the significance of metabolic health and elevated TyG-i levels could encourage early evaluation and engagement in risk-reducing behaviors.

The precise mechanisms underlying the U-shaped relationship between the TyG-i and the risk of developing diabetes in individuals with MASLD are still not fully understood. There is a notable positive association between higher TyG-i values and diabetes, likely linked to insulin resistance. Persistently high TG levels intensify liver fat accumulation, causing increased hepatic triglyceride production and worsening insulin sensitivity (32). This metabolic disturbance enhances lipogenesis, which further reduces insulin’s effectiveness in managing glucose metabolism and increases liver lipid accumulation, eventually damaging pancreatic beta-cell functionality (33). The build-up of lipid droplets within pancreatic islets disrupts glucose-induced insulin release, leading to diabetes onset (34, 35). Moreover, low TyG-i levels are similarly linked to an increased risk of developing diabetes. Interestingly, Black individuals exhibit unexpectedly low TG levels despite high insulin resistance or risk factors for diabetes, a phenomenon potentially explained by the inhibition of insulin-sensitive lipase activity and the consequent reduction in free fatty acid release from fat tissue due to hyperinsulinemia (36–39). Additionally, those with the PNPLA3 148M allele have lower triglyceride levels, increased insulin resistance, and greater vulnerability to diabetes (40). Pancreatic α-cells are vital for maintaining glucose, amino acid, and lipid balance (41). Malfunctions in these α-cells can result in hypoglycemia, which may indicate α-cell dysregulation, a core pathogenic process in diabetes development (42).

Our study is limited to a Japanese cohort, which may constrain the generalizability of our findings. It is essential to consider how genetic, dietary, and healthcare system differences might influence the observed associations between the TyG-i and the risk of T2D. Genetic predispositions play a significant role in metabolic regulation and the pathogenesis of diabetes. Ethnic variations in genes related to lipid metabolism and insulin sensitivity could modulate the relationship between the TyG-i and diabetes risk. For example, certain genetic polymorphisms prevalent in Asian populations may impact triglyceride levels and glucose homeostasis, potentially yielding risk profiles distinct from those in other ethnic groups (43). The traditional Japanese diet—characterized by high consumption of rice, fish, and soy products—imposes unique metabolic effects (44). Dietary patterns may interact with genetic factors to influence TyG-i levels and their associations with diabetes risk. Notably, omega-3 fatty acids abundant in fish have been documented to improve insulin sensitivity, which could affect metabolic outcomes within our cohort (45). Recognizing dietary variations across populations is critical when interpreting our results, as these differences could inform culturally tailored dietary recommendations for T2D prevention. Moreover, the Japanese healthcare system, with its emphasis on universal coverage and preventive care, may significantly impact the management of metabolic diseases (46, 47). Routine health screenings and early interventions are commonplace in Japan, potentially facilitating better management of conditions associated with the TyG-i, such as MASLD. This proactive healthcare approach may alter the observed relationship between TyG-i and diabetes risk, underscoring the need for caution when extrapolating our findings to populations with differing healthcare infrastructures. In light of these considerations, we stress the importance of further research involving diverse populations to validate the U-shaped association between the TyG-i and T2D risk. Future investigations should include a broad range of ethnic groups to examine the consistency and applicability of these findings across varied demographic and clinical contexts.

This study offers several notable advantages. Firstly, we identified a U-shaped association, allowing us to pinpoint the optimal inflection point where the TyG-i affects T2D risk. Secondly, we applied rigorous statistical adjustments to our results to reduce confounding factors, thereby enhancing their validity. Lastly, we employed a diverse array of sensitivity analyses to bolster the validity and reliability of our results, thereby enhancing the overall methodological strength of the study.

Despite these strengths, several limitations warrant consideration. Primarily, the research focused on a Japanese cohort, which may restrict the applicability of the results to other ethnic and geographic populations. Additionally, the definition of T2D employed in this study did not incorporate oral glucose tolerance testing, potentially resulting in an underestimation of T2D incidence. Secondly, although we have controlled for known confounding variables, the possibility remains that unmeasured factors—such as certain lifestyle habits or genetic predispositions—may have influenced the observed relationship between the TyG-i and T2D. Nevertheless, the calculated E-value of 2.32 exceeds the relative risk of 1.78 associated with both TyG-i and potential unknown confounders, implying that the effect of such unmeasured variables on this association is likely minimal. In future prospective investigations, we will strive to systematically collect and incorporate comprehensive information on lifestyle and genetic factors to further validate and strengthen our results. Thirdly, the absence of repeated measurements of the TyG-i precluded the assessment of the impact of longitudinal dynamic changes in TyG-i on T2D risk. The TyG-i, like other metabolic markers, is subject to fluctuations influenced by various factors, including dietary habits, physical activity, weight changes, and underlying metabolic conditions. These dynamic changes may significantly impact an individual’s risk profile for T2D. Incorporating analyses of TyG-i variability over time could enhance our understanding of its relationship with diabetes risk. In light of these considerations, we plan to design future studies to investigate the relationship between changes in the TyG-i and diabetes prognosis.

## Conclusion

This research revealed a U-shaped relationship between the TyG-i and the risk of T2D in adults with MASLD. These results underscore that early intervention using the TyG-i may effectively improve the risk of T2D in patients with MASLD.

## Data Availability

The datasets presented in this study can be found in online repositories. The names of the repository/repositories and accession number(s) can be found below: https://datadryad.org/stash/dataset/doi:10.5061%2Fdryad.8q0p192.
