# Supplementary material for: U-shaped relationship between the triglyceride glucose index and the risk of incident diabetes among MASLD adults: a retrospective cohort study
Source: Front Endocrinol (Lausanne). 2025 Aug 22;16:1516187. doi: 10.3389/fendo.2025.1516187 (PMC12411149; doi:10.3389/fendo.2025.1516187)
Supplement: Supplementary file 1 [file DataSheet1.docx]

**Table S1** The results of the collinearity screening

|  | Step 1 | Step 2 |
| --- | --- | --- |
| **Sex** | 1.5 | 1.5 |
| **Age** | 1.2 | 1.2 |
| **Alcoholic intake** | 1.2 | 1.2 |
| **Smoking status** | 1.2 | 1.2 |
| **Exercise habits** | 1.0 | 1.0 |
| **SBP (mmHg)** | 5.6 | 1.2 |
| **DBP (mmHg)** | 5.7 | NA |
| **BMI (kg/m^2^)** | 1.3 | 1.3 |
| **ALT (IU/L)** | 3.7 | 3.7 |
| **AST (IU/L)** | 3.3 | 3.2 |
| **GGT (IU/L)** | 1.4 | 1.4 |
| **HDL-C (mg/dL)** | 1.3 | 1.3 |
| **TC (mg/dL)** | 1.1 | 1.1 |
| **HbA1c (%)** | 1.1 | 1.1 |

**Table S2A** The Baseline Characteristics of male participants

| **LAP** | Q1 (LAP≤7.08) | Q2 (7.08< LAP ≤13.81) | Q3 (13.81< LAP ≤25.68) | Q4 (LAP>25.68) | P-value |
| --- | --- | --- | --- | --- | --- |
| **Participants** | 1992 | 1986 | 1998 | 1993 |  |
| **Age(years)** | 41.04 ± 8.06 | 43.25 ± 8.00 | 44.25 ± 7.83 | 44.16 ± 7.62 | <0.001 |
| **Alcoholic intake (g/wk)** | 18 (1-99.3) | 35 (1-126) | 22 (1-126) | 36 (1-126) | <0.001 |
| **Smoking status** |  |  |  |  | <0.001 |
| Never-smoker | 800 (40.16%) | 722 (36.35%) | 624 (31.23%) | 612 (30.71%) |  |
| Ex-smoker | 520 (26.10%) | 574 (28.90%) | 630 (31.53%) | 580 (29.10%) |  |
| Current-smoker | 672 (33.73%) | 690 (34.74%) | 744 (37.24%) | 801 (40.19%) |  |
| **Exercise habits** |  |  |  |  | <0.001 |
| No | 1540 (77.31%) | 1579 (79.51%) | 1672 (83.68%) | 1724 (86.50%) |  |
| Yes | 452 (22.69%) | 407 (20.49%) | 326 (16.32%) | 269 (13.50%) |  |
| **SBP (mmHg)** | 112.91 ± 12.33 | 117.18 ± 12.84 | 119.98 ± 13.46 | 124.95 ± 14.61 | <0.001 |
| **DBP (mmHg)** | 70.48 ± 9.01 | 73.67 ± 9.13 | 75.88 ± 9.49 | 79.44 ± 9.99 | <0.001 |
| **BMI (kg/m^2^)** | 20.48 ± 1.80 | 22.40 ± 1.84 | 23.80 ± 2.13 | 25.82 ± 2.91 | <0.001 |
| **WC (cm)** | 72.50 ± 4.11 | 78.76 ± 4.16 | 82.98 ± 4.89 | 88.44 ± 6.74 | <0.001 |
| **ALT (IU/L)** | 16 (13-21) | 19 (15-25) | 22 (17-30) | 27 (21-39) | <0.001 |
| **AST (IU/L)** | 17 (14-21) | 18 (14-21) | 19 (15-23) | 21 (17-26) | <0.001 |
| **GGT (IU/L)** | 15 (13-20) | 18 (14-25) | 21 (16-30) | 27 (20-41) | <0.001 |
| **HDL-C (mmol/L)** | 1.52 ± 0.37 | 1.36 ± 0.32 | 1.23 ± 0.29 | 1.09 ± 0.24 | <0.001 |
| **TG (mmol/L)** | 0.53 (0.42-0.69) | 0.76 (0.62-0.93) | 1.07 (0.88-1.31) | 1.78 (1.42-2.33) | <0.001 |
| **TC (mmol/L)** | 4.77 ± 0.77 | 5.04 ± 0.79 | 5.26 ± 0.78 | 5.56 ± 0.87 | <0.001 |
| **HbA1c (%)** | 5.11 ± 0.29 | 5.12 ± 0.30 | 5.17 ± 0.33 | 5.23 ± 0.34 | <0.001 |
| **FPG (mmol/L)** | 5.18 ± 0.37 | 5.28 ± 0.35 | 5.34 ± 0.35 | 5.43 ± 0.35 | <0.001 |
| **DM** |  |  |  |  | <0.001 |
| No | 1968 (98.80%) | 1952 (98.29%) | 1946 (97.40%) | 1837 (92.17%) |  |
| Yes | 24 (1.20%) | 34 (1.71%) | 52 (2.60%) | 156 (7.83%) |  |
| **Cumulative incidence** (%) (95% CI) | 1.20 (0.73-1.68) | 1.71 (1.14-2.28) | 2.60 (1.90-3.30) | 7.83 (6.65-9.01) | <0.001 |
| **Per 100,000 person-year** | 202.93 | 274.12 | 406.33 | 1224.12 | <0.001 |

Values are presented as n (%) or mean ± SD or median (quartile)

LAP: lipid accumulation product; BMI: body mass index; WC: waist circumference; SBP: systolic blood pressure; DBP: diastolic blood pressure; ALT: alanine aminotransferase; AST: aspartate aminotransferase; GGT: gamma-glutamyl transferase; HDL-C: high-density lipoprotein cholesterol; TC: total cholesterol; TG: triglycerides; HbA1c: hemoglobin A1c; FPG: fasting plasma glucose; CI: confidence interval; DM: diabetes mellitus.

**Table S2B** The Baseline Characteristics of female participants

| **LAP** | Q1 (LAP≤3.55) | Q2 (3.55< LAP ≤6.66) | Q3 (6.66< LAP ≤12.48) | Q4 (LAP>12.48) | P-value |
| --- | --- | --- | --- | --- | --- |
| **Participants** | 1688 | 1688 | 1689 | 1690 |  |
| **Age(years)** | 39.61 ± 7.72 | 41.32 ± 7.70 | 43.55 ± 7.73 | 46.42 ± 7.72 | <0.001 |
| **Alcoholic intake (g/wk)** | 1 (0-2.8) | 1 (0-12) | 1 (0-12) | 1 (0-2.8) | 0.004 |
| **Smoking status** |  |  |  |  | 0.023 |
| Never-smoker | 1493 (88.45%) | 1486 (88.03%) | 1453 (86.03%) | 1452 (85.92%) |  |
| Ex-smoker | 96 (5.69%) | 115 (6.81%) | 115 (6.81%) | 106 (6.27%) |  |
| Current-smoker | 99 (5.86%) | 87 (5.15%) | 121 (7.16%) | 132 (7.81%) |  |
| **Exercise habits** |  |  |  |  | 0.037 |
| No | 1399 (82.88%) | 1446 (85.66%) | 1421 (84.13%) | 1455 (86.09%) |  |
| Yes | 289 (17.12%) | 242 (14.34%) | 268 (15.87%) | 235 (13.91%) |  |
| **SBP (mmHg)** | 103.72 ± 11.86 | 106.25 ± 12.21 | 109.42 ± 13.07 | 117.45 ± 15.46 | <0.001 |
| **DBP (mmHg)** | 64.23 ± 8.14 | 65.60 ± 8.60 | 67.54 ± 9.19 | 72.92 ± 10.53 | <0.001 |
| **BMI (kg/m^2^)** | 18.83 ± 1.57 | 20.09 ± 1.68 | 21.35 ± 2.00 | 23.94 ± 3.23 | <0.001 |
| **WC (cm)** | 64.24 ± 3.71 | 69.12 ± 3.89 | 73.43 ± 4.80 | 80.55 ± 7.23 | <0.001 |
| **ALT (IU/L)** | 13 (10-16) | 13 (11-16) | 13 (10-17) | 15 (12-20) | <0.001 |
| **AST (IU/L)** | 16 (13-19) | 15 (13-18) | 16 (13-19) | 17 (14-20) | <0.001 |
| **GGT (IU/L)** | 11 (9-13) | 11 (9-14) | 12 (10-14) | 13 (11-17) | <0.001 |
| **HDL-C (mmol/L)** | 1.78 ± 0.37 | 1.73 ± 0.37 | 1.64 ± 0.37 | 1.44 ± 0.34 | <0.001 |
| **TG (mmol/L)** | 0.36 (0.27-0.47) | 0.46 (0.37-0.60) | 0.61 (0.50-0.75) | 0.97 (0.78-1.26) | <0.001 |
| **TC (mmol/L)** | 4.73 ± 0.78 | 4.91 ± 0.78 | 5.12 ± 0.85 | 5.50 ± 0.88 | <0.001 |
| **HbA1c (%)** | 5.10 ± 0.30 | 5.12 ± 0.30 | 5.18 ± 0.31 | 5.31 ± 0.33 | <0.001 |
| **FPG (mmol/L)** | 4.86 ± 0.36 | 4.91 ± 0.37 | 5.00 ± 0.37 | 5.17 ± 0.39 | <0.001 |
| **DM** |  |  |  |  | <0.001 |
| No | 1683 (99.70%) | 1682 (99.64%) | 1680 (99.47%) | 1629 (96.39%) |  |
| Yes | 5 (0.30%) | 6 (0.36%) | 9 (0.53%) | 61 (3.61%) |  |
| **Cumulative incidence** (%) (95% CI) | 0.30 (0.04-0.56) | 0.36 (0.07-0.64) | 0.53 (0.19-0.88) | 3.61 (2.72-4.50) | <0.001 |
| **Per 100,000 person-year** | 47.06 | 59.31 | 92.57 | 645.69 | <0.001 |

Values are presented as n (%) or mean ± SD or median (quartile)

LAP: lipid accumulation product; BMI: body mass index; WC: waist circumference; SBP: systolic blood pressure; DBP: diastolic blood pressure; ALT: alanine aminotransferase; AST: aspartate aminotransferase; GGT: gamma-glutamyl transferase; HDL-C: high-density lipoprotein cholesterol; TC: total cholesterol; TG: triglycerides; HbA1c: hemoglobin A1c; FPG: fasting plasma glucose.

**Table S3** The results of the univariate analysis results of risk factors associated with diabetes

|  | **N (%)/M±SD** | **HR (95% CI)** | ***P* value** |
| --- | --- | --- | --- |
| **Sex** |  |  | <0.0001 |
| Female | 6755 (45.88%) | ref |  |
| Male | 7969 (54.12%) | 2.54 (1.98, 3.26) |  |
| **Age(years)** | 42.97 ± 8.05 | 1.06 (1.04, 1.07) | <0.0001 |
| **Alcoholic intake (g/wk)** | 47.34 ± 82.03 | 1.00 (1.00, 1.00) | 0.0091 |
| **Smoking status** |  |  |  |
| Never-smoker | 8642 (58.69%) | ref |  |
| Ex-smoker | 2736 (18.58%) | 1.74 (1.31, 2.32) | 0.0002 |
| Current-smoker | 3346 (22.72%) | 2.67 (2.11, 3.39) | <0.0001 |
| **Exercise habits** |  |  | 0.0574 |
| No | 12236 (83.10%) | ref |  |
| Yes | 2488 (16.90%) | 0.74 (0.54, 1.01) |  |
| **SBP (mmHg)** | 114.38 ± 14.89 | 1.03 (1.03, 1.04) | <0.0001 |
| **DBP (mmHg)** | 71.52 ± 10.51 | 1.05 (1.04, 1.06) | <0.0001 |
| **BMI (kg/m^2^)** | 22.18 ± 3.11 | 1.25 (1.22, 1.27) | <0.0001 |
| **WC (cm)** | 76.62 ± 8.96 | 1.10 (1.09, 1.11) | <0.0001 |
| **ALT (IU/L)** | 20.06 ± 14.52 | 1.01 (1.01, 1.01) | <0.0001 |
| **AST (IU/L)** | 18.32 ± 8.68 | 1.01 (1.01, 1.01) | <0.0001 |
| **GGT (IU/L)** | 20.31 ± 18.18 | 1.01 (1.01, 1.01) | <0.0001 |
| **HDL-C (mmol/L)** | 1.46 ± 0.40 | 0.15 (0.11, 0.21) | <0.0001 |
| **TG (mmol/L)** | 0.91 ± 0.65 | 1.81 (1.69, 1.94) | <0.0001 |
| **TC (mmol/L)** | 5.12 ± 0.86 | 1.50 (1.35, 1.68) | <0.0001 |
| **HbA1c (%)** | 5.17 ± 0.32 | 56.41 (40.41, 78.74) | <0.0001 |
| **FPG (mmol/L)** | 5.16 ± 0.41 | 28.03 (20.35, 38.60) | <0.0001 |
| **LAP** | 15.23 ± 16.60 | 1.03 (1.03, 1.03) | <0.0001 |

N (%)/M±SD: Values are presented n (%) or mean ± SD

LAP: lipid accumulation product; BMI: body mass index; WC: waist circumference; SBP: systolic blood pressure; DBP: diastolic blood pressure; ALT: alanine aminotransferase; AST: aspartate aminotransferase; GGT: gamma-glutamyl transferase; HDL-C: high-density lipoprotein cholesterol; TC: total cholesterol; TG: triglycerides; HbA1c: hemoglobin A1c; FPG: fasting plasma glucose; HR: hazard ratio; CI: confidence interval; Ref: Reference.

**Table S4** Relationship between LAP and incident diabetes in different sensitivity analyses

| Variable | Model 5 (HR,95%CI, P) | Model 6 (HR,95% CI, P) |
| --- | --- | --- |
| **Total**  LAP | 1.01 (1.01, 1.02) 0.0001 | 1.01 (1.00, 1.02) 0.0111 |
| **Total**  LAP (quartile) |  |  |
| Q1 | Ref. | Ref. |
| Q2 | 0.66 (0.38, 1.16) 0.1465 | 0.90 (0.36, 2.23) 0.8211 |
| Q3 | 0.59 (0.34, 1.01) 0.0555 | 1.12 (0.49, 2.57) 0.7897 |
| Q4 | 0.73 (0.40, 1.33) 0.2978 | 1.25 (0.51, 3.06) 0.6245 |
| P for trend | 0.7000 | 0.4313 |
| **Female**  LAP | 1.02 (1.01, 1.03) 0.0041 | 1.00 (0.99, 1.02) 0.6692 |
| **Female**  LAP (quartile) |  |  |
| Q1 | Ref. | Ref. |
| Q2 | 0.98 (0.29, 3.23) 0.9683 | 1.90 (0.34, 10.50) 0.4641 |
| Q3 | 0.91 (0.29, 2.82) 0.8705 | 1.38 (0.26, 7.47) 0.7080 |
| Q4 | 2.14 (0.71, 6.43) 0.1761 | 2.72 (0.49, 15.03) 0.2501 |
| P for trend | 0.0797 | 0.2749 |
| **Male**  LAP | 1.01 (1.00, 1.02) 0.0039 | 1.01 (1.00, 1.02) 0.0228 |
| **Male**  LAP (quartile) |  |  |
| Q1 | Ref. | Ref. |
| Q2 | 0.69 (0.39, 1.24) 0.2130 | 1.52 (0.54, 4.29) 0.4291 |
| Q3 | 0.61 (0.34, 1.10) 0.1016 | 1.47 (0.53, 4.06) 0.4598 |
| Q4 | 0.91 (0.48, 1.71) 0.7660 | 1.86 (0.65, 5.30) 0.2457 |
| P for trend | 0.6320 | 0.2524 |

Model 5 was sensitivity analysis after excluding those with elevated blood pressure (SBP ≥140 mmHg or DBP ≥90 mmHg). We adjusted sex, age, alcoholic intake, smoking status, exercise habits, BMI, ALT, AST, GGT, HDL-C, TC, and FPG.

Model 6 was sensitivity analysis after excluding those with age≥45 years. We adjusted sex, age, alcoholic intake, smoking status, exercise habits, BMI, ALT, AST, GGT, HDL-C, TC, and FPG.

Note: The models were not adjusted for sex variables in both male and female models.

HR: hazard ratio; CI: confidence interval; Ref.: Reference; LAP: lipid accumulation product.

**Table S5** Effect size of LAP on diabetes in prespecified and exploratory subgroups

| Characteristic | No of patients | Effect size(95%CI) | P value | P for interaction |
| --- | --- | --- | --- | --- |
| **Sex** |  |  |  | 0.2126 |
| Female | 6755 | 1.01 (1.00, 1.02) | 0.0073 |  |
| Male | 7969 | 1.01 (1.00, 1.01) | 0.0118 |  |
| **Alcoholic intake (g/wk)** |  |  |  | 0.0083 |
| =0 | 4470 | 1.02 (1.01, 1.03) | <0.0001 |  |
| >0 | 10254 | 1.00 (1.00, 1.01) | 0.2558 |  |
| **Smoking status** |  |  |  | 0.8655 |
| Never-smoker | 8642 | 1.01 (1.00, 1.02) | 0.0605 |  |
| Ex-smoker | 2736 | 1.01 (1.00, 1.02) | 0.0996 |  |
| Current-smoker | 3346 | 1.01 (1.00, 1.01) | 0.1317 |  |
| **Exercise habits** |  |  |  | 0.8313 |
| No | 12236 | 1.01 (1.00, 1.01) | 0.0070 |  |
| Yes | 2488 | 1.01 (1.00, 1.02) | 0.1961 |  |
| **SBP (mmHg)** |  |  |  | 0.0558 |
| <140 | 13993 | 1.01 (1.01, 1.02) | 0.0002 |  |
| ≥140 | 731 | 1.00 (0.98, 1.01) | 0.6591 |  |
| **DBP (mmHg)** |  |  |  | 0.0451 |
| <90 | 14002 | 1.01 (1.00, 1.02) | 0.0003 |  |
| ≥90 | 722 | 1.00 (0.98, 1.01) | 0.5021 |  |

Note 1: The above model adjusted for we adjusted forsex, age, alcoholic intake, smoking status, exercise habits, BMI, ALT, AST, GGT, HDL-C, TC, and FPG.

Note 2: The model is not adjusted for the stratification variable in each case.

**Table S6** Relationship between LAP and incident diabetes in participants of different ages.

| Variable | Model 7 (HR,95%CI, P) | Model 8 (HR,95% CI, P) | Model 9 (HR,95% CI, P) | Model 10 (HR,95% CI, P) |
| --- | --- | --- | --- | --- |
| **Total**  LAP | 1.03 (1.02, 1.03) <0.0001 | 1.03 (1.02, 1.03) <0.0001 | 1.02 (1.01, 1.02) <0.0001 | 1.01 (1.00, 1.01) 0.0042 |
| **Total**  LAP (quartile) |  |  |  |  |
| Q1 | Ref. | Ref. | Ref. | Ref. |
| Q2 | 1.35 (0.80, 2.29) 0.2631 | 1.24 (0.73, 2.11) 0.4202 | 0.86 (0.50, 1.46) 0.5760 | 0.67 (0.39, 1.14) 0.1416 |
| Q3 | 2.53 (1.57, 4.07) 0.0001 | 2.17 (1.34, 3.49) 0.0015 | 1.10 (0.67, 1.81) 0.7019 | 0.65 (0.39, 1.09) 0.1008 |
| Q4 | 7.87 (5.07, 12.21) <0.0001 | 6.46 (4.15, 10.04) <0.0001 | 2.02 (1.23, 3.33) 0.0059 | 0.78 (0.45, 1.36) 0.3858 |
| P for trend | <0.0001 | <0.0001 | <0.0001 | 0.9108 |
| **Young individuals**  LAP | 1.03 (1.03, 1.04) <0.0001 | 1.03 (1.03, 1.03) <0.0001 | 1.02 (1.01, 1.02) <0.0001 | 1.01 (1.00, 1.02) 0.0111 |
| LAP (quartile) |  |  |  |  |
| Q1 | Ref. | Ref. | Ref. | Ref. |
| Q2 | 1.82 (0.74, 4.44) 0.1908 | 1.75 (0.72, 4.29) 0.2190 | 1.21 (0.49, 2.97) 0.6801 | 0.90 (0.36, 2.23) 0.8211 |
| Q3 | 5.18 (2.38, 11.27) <0.0001 | 4.68 (2.14, 10.25) 0.0001 | 2.23 (1.00, 4.97) 0.0501 | 1.12 (0.49, 2.57) 0.7897 |
| Q4 | 17.65 (8.60, 36.25) <0.0001 | 15.87 (7.64, 32.98) <0.0001 | 4.15 (1.85, 9.28) 0.0005 | 1.25 (0.51, 3.06) 0.6245 |
| P for trend | <0.0001 | <0.0001 | <0.0001 | 0.4313 |
| **Middle-aged individuals**  LAP | 1.03 (1.02, 1.03) <0.0001 | 1.02 (1.02, 1.03) <0.0001 | 1.01 (1.01, 1.02) <0.0001 | 1.01 (1.00, 1.01) 0.0459 |
| LAP (quartile) |  |  |  |  |
| Q1 | Ref. | Ref. | Ref. | Ref. |
| Q2 | 1.00 (0.52, 1.91) 0.9895 | 0.92 (0.48, 1.77) 0.8079 | 0.67 (0.34, 1.29) 0.2303 | 0.54 (0.27, 1.06) 0.0743 |
| Q3 | 1.37 (0.76, 2.47) 0.2997 | 1.23 (0.68, 2.23) 0.5006 | 0.69 (0.37, 1.30) 0.2548 | 0.49 (0.25, 0.95) 0.0355 |
| Q4 | 4.21 (2.50, 7.09) <0.0001 | 3.47 (2.04, 5.90) <0.0001 | 1.33 (0.71, 2.51) 0.3776 | 0.66 (0.32, 1.36) 0.2589 |
| P for trend | <0.0001 | <0.0001 | 0.0284 | 0.97 (0.77, 1.22) 0.7932 |

Model 7: we did not adjust for any covariants.

Model 8: we adjusted for sex, age, alcoholic intake, smoking status, and exercise habits.

Model 9: we adjusted for sex, age, alcoholic intake, smoking status, exercise habits, and BMI.

Model 10: we adjusted for sex, age, alcoholic intake, smoking status, exercise habits, BMI, ALT, AST, GGT, HDL-C, TC, and FPG.

HR: hazard ratio; CI: confidence interval; Ref.: Reference; LAP: lipid accumulation product.

**Figure S1** Directed Acyclic Graph of potential confounders of the association between LAP and the risk of DM

**
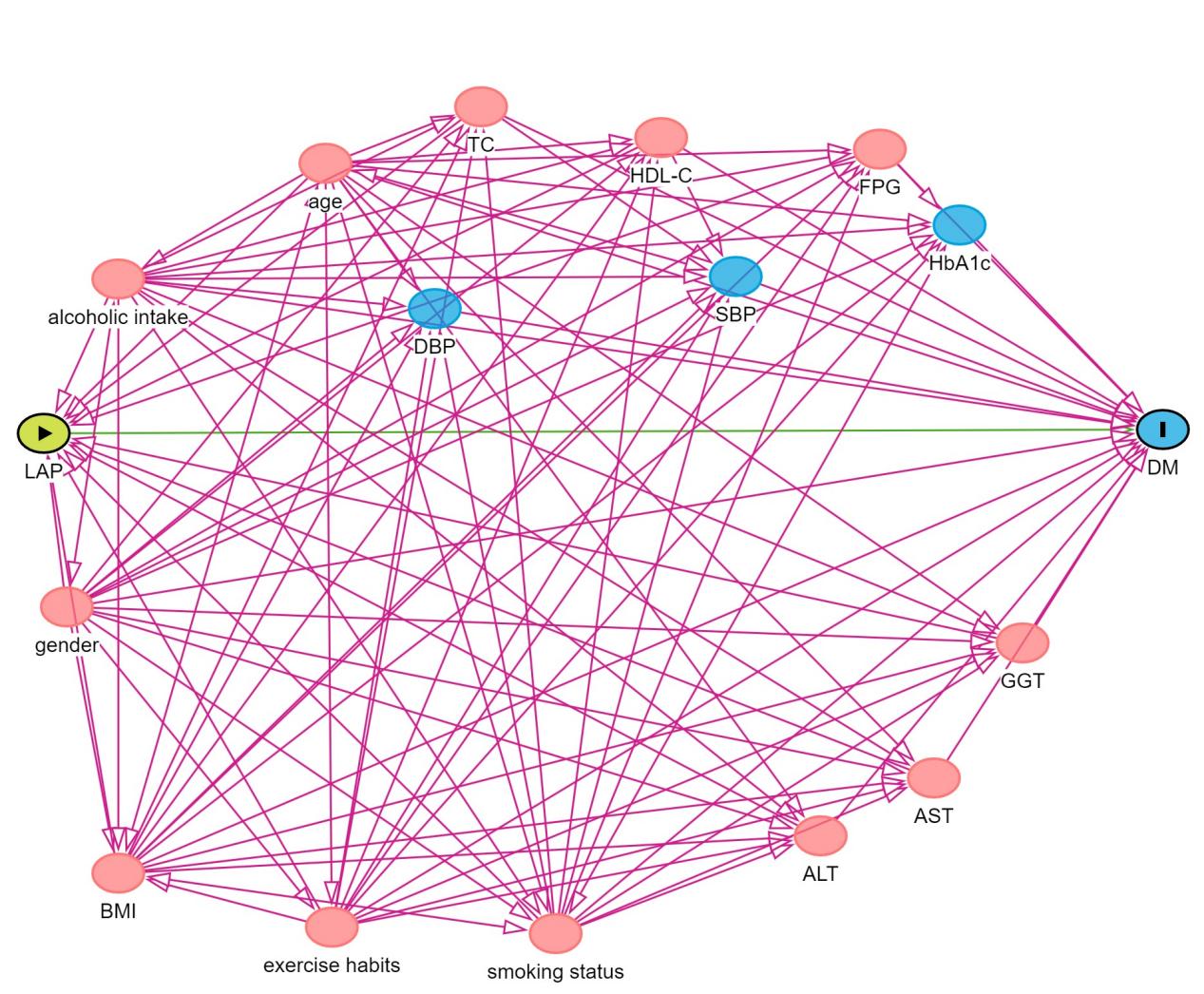
**
